# Sociodemographic Disparities in the Diagnosis and Prognosis of Patients With Cervical Cancer: An Analysis of the Surveillance, Epidemiology, and End Results Program

**DOI:** 10.7759/cureus.41477

**Published:** 2023-07-06

**Authors:** Jad Zreik, Maya Asami Takagi, Maheen F Akhter, Amna A Ahmad, Kush Pandya, Jasmine M Madoun, Beth Bailey

**Affiliations:** 1 Internal Medicine, Central Michigan University College of Medicine, Saginaw, USA; 2 Surgery, Central Michigan University College of Medicine, Mt. Pleasant, USA; 3 Health Services Research, Central Michigan University College of Medicine, Saginaw, USA

**Keywords:** prognosis, hysterectomy, disparities, socioeconomic status, ethnicity, race, cervical cancer

## Abstract

Background

While the incidence and mortality rates of cervical cancer are declining due to improved prevention, screening, and treatment, inequitable access to care may contribute to worse patient outcomes. Therefore, we sought to evaluate sociodemographic disparities in the diagnosis and prognosis of patients with cervical cancer.

Methodology

The Surveillance, Epidemiology, and End Results (SEER) database was queried for adult women diagnosed with cervical cancer from 2010 to 2015. Sociodemographic groups of interest included patient race/ethnicity (non-Hispanic White/Hispanic White/Black/Other), residential setting (rural/urban), and county median household income (<$45,000/$45,000-59,999/$60,000-74,999/≥$75,000). Outcomes of interest included stage at diagnosis, receipt of hysterectomy, and overall survival (OS). Outcomes were evaluated using Pearson’s chi-square test, multivariable logistic regression, and multivariable Cox proportional hazards.

Results

A total of 5,726 patients were identified with an average age of 50.1 years (SD = 14.6). Significant differences in cancer stage at diagnosis were identified based on race/ethnicity (p < 0.001) and household income (p = 0.012). On adjusted analysis, Black patients were found to be significantly less likely to receive a hysterectomy compared to non-Hispanic White patients (odds ratio (OR) = 0.46; 95% confidence interval (CI) = 0.37-0.56). Lower household income was associated with poorer survival for stage I (<$45,000 vs. >$75,000: hazard ratio (HR) = 1.53; 95% interquartile range (IQR) = 1.00-2.33), II ($45,000-59,999 vs. >$75,000: HR = 1.67; 95% IQR = 1.19-2.35), and IV (<$45,000 vs. >$75,000: HR = 1.64; 95% IQR = 1.22-2.29) disease. Black race was associated with poorer OS for stage IV disease (HR = 1.29; 95% IQR = 1.06-1.56).

Conclusions

This study highlights significant disparities in disease progression at diagnosis and OS for cervical cancer patients based on race/ethnicity and household income. These findings may assist policymakers in developing strategies for mitigating these disparities.

## Introduction

The incidence of cervical cancer, previously the leading cause of cancer death for women in the United States, has decreased dramatically with the advent of cervical cancer screening [1]. Regular Papanicolaou tests as part of routine ambulatory care visits have decreased the mortality rate of cervical cancer [1-3]. The introduction of human papillomavirus (HPV) testing and vaccination has also contributed to decreased HPV-associated incidence of the disease [4,5]. Despite these advances, disparities in cervical cancer incidence and mortality in the United States remain [1].

It has been suggested in the medical literature that social determinants of health strongly influence cervical cancer outcomes [6]. Social determinants of health include race/ethnicity, age, socioeconomic status (SES), education level, and residential zip code [7]. Women in rural areas may experience limited access to preventative health care, such as cervical cancer screening, making progression to malignancy more likely [8]. They may also suffer from higher rates of cervical cancer incidence as well as mortality when compared to women in urban areas [9]. Racial/ethnic disparities have also been shown to exist among HPV diagnoses, which predisposes patients to developing cervical cancer [10]. Individuals with lower SES have lower rates of cervical cancer screening and, thus, higher rates of cervical cancer in comparison to those with higher SES [11]. Additionally, in the current literature, inequitable receipt of a hysterectomy has been shown to further contribute to underestimations of disparities in cervical cancer incidence and mortality [12,13]. Therefore, the objective of this study was to further examine the effects of patient residential region, SES, and race/ethnicity on the diagnosis and prognosis of cervical cancer using a national, population-based oncology database.

## Materials and methods

Patient population

The Surveillance, Epidemiology, and End Results (SEER) program database was utilized for this study. SEER is sponsored by the National Cancer Institute and is a population-based registry providing a representative sample of geographic regions and subpopulations of oncology patients in the United States. Overall, it captures approximately 30% of cancer diagnoses in the United States along with information on patient demographics, treatment, and survival [14]. Given the deidentified nature of the database, this study was exempt from local Institutional Review Board approval.

**Figure 1:**
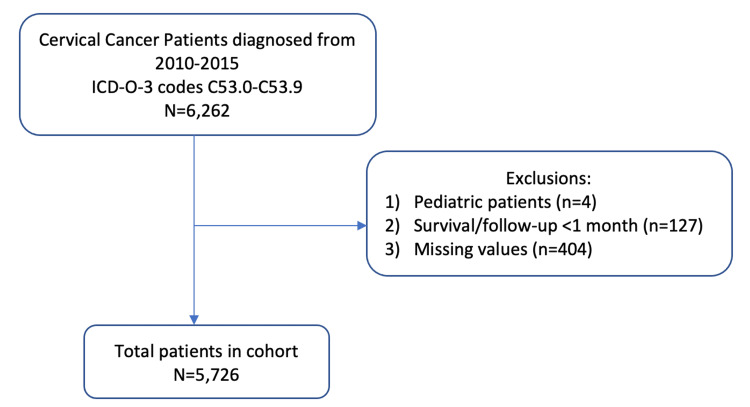
Flowchart for patient selection.

Women with cervical cancer were identified using the International Classification of Diseases for Oncology, third revision (ICD-O-3) topography codes C53.0-C53.9. Patients were further included in the study if they were adults (age ≥18) diagnosed from 2010 to 2015. Those with an overall survival (OS) of less than a month were excluded to mitigate immortal time bias [15]. The patient selection method is summarized in Figure 1.

Predictors and outcomes of interest

Patient demographics included age at diagnosis, race/ethnicity, residential region, county-level median income, and cancer stage at diagnosis. Race/ethnicity was classified as non-Hispanic White, Hispanic White, Black, or other. The residential region was classified as either rural or urban/suburban according to the designated Rural-Urban Continuum code for each patient’s zip code [16]. Patient income was set according to the average household income in the patient’s zip code of residence and categorized as <$45,000, $45,000-59,999, $60,000-74,999, or ≥$75,000. Outcomes of interest included receipt of a hysterectomy and OS. OS was defined as the time, in months, until death or last follow-up.

Statistical analysis

Characteristics of the patient population were first described using frequencies with proportions for categorical variables and means with standard deviations (SD) for continuous variables. Characteristics and outcomes were then compared between patient residential region, race/ethnicity, and median county household income. Characteristics and outcomes were compared between these demographic groups using the two-sample t-test for continuous variables and the chi-square test for categorical variables. OS for each demographic group was analyzed using the Kaplan-Meier method and compared using the log-rank test. Given the significant influence of cancer stage on prognosis, each demographic group was stratified by cancer stage for Kaplan-Meier and log-rank analyses.

Univariate and multivariable logistic regression were used to evaluate the unadjusted and adjusted association, respectively, of patient demographics with receipt of a hysterectomy. The variance inflation factor was used to assess collinearity between covariates in the multivariable model. Univariate and multivariable Cox proportional hazards regression was used to evaluate the unadjusted and adjusted association, respectively, of patient demographics with OS. The proportional hazards assumption was checked by evaluating scaled Schoenfeld residuals. As the staging variable demonstrated a significant, non-random association with time, these models were stratified by cancer stage to meet the proportional hazards assumption. For all regression models, only variables significant at p < 0.10 on the initial univariate analysis were included in the subsequent multivariable regression models.

Analysis was performed using R version 4.0.5 (R Foundation for Statistical Computing, Vienna, Austria). P-values were two-sided and values <0.05 were considered statistically significant.

## Results

Patient characteristics

A total of 5,726 women diagnosed with cervical cancer comprised the sample. Their median age was 48 years (interquartile range (IQR) = 39-60), with the majority identifying as non-Hispanic White (58.9%), followed by Hispanic White (14%), Black (13.7%), and other race/ethnicity (13.4%). Most identified patients resided in an urban/suburban region (89%) compared to a rural region (11%). Additionally, patients most often resided in counties with a median household income of $60,000-74,999 (35%) or ≥$75,000 (34.3%), followed by an income of $45,000-59,999 (23.1%) and <$45,000 (7.7%). Patients were most often diagnosed with Stage I disease (50.3%), followed by Stage III (21.4%), Stage IV (15.5%), and Stage II (12.8%). A hysterectomy was performed in 45.7% (n = 2,614) of cases. Median OS was 50 months (IQR = 24-76) for all patients (Table 1).

**Table 1:** Patient characteristics.

	Total (N = 5,726)
Age, mean (SD)	50.1 (14.6)
Race/Ethnicity, n (%)	
Non-Hispanic White	3,373 (58.9%)
Hispanic White	803 (14.0%)
Black	782 (13.7%)
Other	768 (13.4%)
Residential region, n (%)	
Urban	5,098 (89.0%)
Rural	628 (11.0%)
County median household income, n (%)	
< $45,000	440 (7.7%)
$45,000–59,999	1,321 (23.1%)
$60,000–74,999	2,003 (35.0%)
≥$75,000	1,962 (34.3%)
Stage, n (%)	
I	2,878 (50.3%)
II	733 (12.8%)
III	1,226 (21.4%)
IV	889 (15.5%)
Hysterectomy, n (%)	
No	3,112 (54.3%)
Yes	2,614 (45.7%)
Survival or last follow-up, median (IQR)	50 (24–76)

Significant differences in disease progression at diagnosis were also identified after stratifying patients by sociodemographic variables. A significant difference in cancer stage at diagnosis was identified based on patient race/ethnicity (p < 0.001), as Black patients were diagnosed with Stage I disease at the lowest rate (37.9%), followed by other race/ethnicity (47.7%), Hispanic White (53.1%, n = 426), and non-Hispanic White (53.1%) patients (Table 2). Similarly, a significant difference in cancer stage was identified based on average county-level household income (p = 0.012), with patients residing in counties with an income <$45,000 diagnosed with stage I disease at the lowest rate (44.5%), followed by $45,000-59,999 (47.0%), $60,000-74,999 (50.6%), and ≥$75,000 (53.4%) (Table 3). In contrast, no significant difference in cervical cancer staging at diagnosis was identified based on patient urban or rural residential region (p = 0.813) (Table 4).

**Table 2:** Patient characteristics stratified by race/ethnicity.

	Non-Hispanic White (N = 3,373)	Hispanic White (N = 803)	Black (N = 782)	Other (N = 768)	P-value
Age, mean (SD)	50.6 (14.6)	51.4 (13.7)	51.4 (14.9)	50.9 (14.8)	<0.001
Residential region, n (%)					<0.001
Urban	2,901 (86.0%)	723 (90.0%)	778 (99.5%)	696 (90.6%)	
Rural	472 (14.0%)	80 (10.0%)	4 (0.5%)	72 (9.4%)	
County median household income, n (%)					<0.001
< $45,000	179 (5.3%)	97 (12.1%)	131 (16.8%)	33 (4.3%)	
$45,000–59,999	825 (24.5%)	183 (22.8%)	233 (29.8%)	80 (10.4%)	
$60,000–74,999	1,356 (40.2%)	211 (26.3%)	289 (37.0%)	147 (19.1%)	
≥$75,000	1,013 (30.0%)	312 (38.9%)	129 (16.5%)	508 (66.1%)	
Stage, n (%)					<0.001
I	1,790 (53.1%)	426 (53.1%)	296 (37.9%)	366 (47.7%)	
II	400 (11.9%)	103 (12.8%)	127 (16.2%)	103 (13.4%)	
III	675 (20.0%)	173 (21.5%)	189 (24.2%)	189 (24.6%)	
IV	508 (15.1%)	101 (12.6%)	170 (21.7%)	110 (14.3%)	
Hysterectomy, n (%)					<0.001
No	1,713 (50.8%)	427 (53.2%)	565 (72.3%)	407 (53.0%)	
Yes	1,660 (49.2%)	376 (46.8%)	217 (27.7%)	361 (47.0%)	
Survival or last follow-up, median (IQR)	53 (29–79)	47 (21–72)	44 (14–68)	52 (24–75)	<0.001

**Table 3:** Patient characteristics stratified by county median household income.

		$45,000–59,999 (N = 1,321)	$60,000–74,999 (N = 2,003)	≥$75,000 (N = 1,962)	P-value
Age, mean (SD)	50.2 (15.3)	49.7 (14.8)	49.9 (14.2)	50.5 (14.8)	0.449
Race/Ethnicity, n (%)					<0.001
Non-Hispanic White	179 (40.7%)	825 (62.5%)	1,356 (67.7%)	1,013 (51.6%)	
Hispanic White	97 (22.0%)	183 (13.9%)	211 (10.5%)	312 (15.9%)	
Black	131 (29.8%)	233 (17.6%)	289 (14.4%)	129 (6.6%)	
Other	33 (7.5%)	80 (6.1%)	147 (7.3%)	508 (25.9%)	
Residential region, n (%)					<0.001
Urban	310 (70.5%)	948 (71.8%)	1,926 (96.2%)	1,914 (97.6%)	
Rural	130 (29.5%)	373 (28.2%)	77 (3.8%)	48 (2.4%)	
Stage, n (%)					0.012
I	196 (44.5%)	621 (47.0%)	1,014 (50.6%)	1,047 (53.4%)	
II	69 (15.7%)	175 (13.2%)	258 (12.9%)	231 (11.8%)	
III	107 (24.3%)	304 (23.0%)	423 (21.1%)	392 (20.0%)	
IV	68 (15.5%)	221 (16.7%)	308 (15.4%)	292 (14.9%)	
Hysterectomy, n (%)					0.001
No	267 (60.7%)	739 (55.9%)	1,098 (54.8%)	1,008 (51.4%)	
Yes	173 (39.3%)	582 (44.1%)	905 (45.2%)	954 (48.6%)	
Survival or last follow-up, median (IQR)	49 (19–67)	50 (20–81)	51 (24–75)	50 (29–78)	<0.001

**Table 4:** Patient characteristics stratified by patient residential region.

	Urban (N = 5,098)	Rural (N = 628)	P-value
Age, mean (SD)	50.1 (14.7)	50.2 (14.7)	0.945
Race/Ethnicity, n (%)			<0.001
Non-Hispanic White	2,901 (56.9%)	472 (75.2%)	
Hispanic White	723 (14.2%)	80 (12.7%)	
Black	778 (15.3%)	4 (0.6%)	
Other	696 (13.7%)	72 (11.5%)	
County median household income, n (%)			<0.001
< $45,000	310 (6.1%)	130 (20.7%)	
$45,000–59,999	948 (18.6%)	373 (59.4%)	
$60,000–74,999	1,926 (37.8%)	77 (12.3%)	
≥$75,000	1,914 (37.5%)	48 (7.6%)	
Stage, n (%)			0.813
I	2,573 (50.5%)	305 (48.6%)	
II	648 (12.7%)	85 (13.5%)	
III	1,090 (21.4%)	136 (21.7%)	
IV	787 (15.4%)	102 (16.2%)	
Hysterectomy, n (%)			0.337
No	2,782 (54.6%)	330 (52.5%)	
Yes	2,316 (45.4%)	298 (47.5%)	
Survival or last follow-up, median (IQR)	50 (24–76)	51.5 (24.8–78)	0.510

Disparities in receipt of hysterectomy

Disparities in the receipt of a hysterectomy were assessed using unadjusted and adjusted logistic regression. Unadjusted ratios showed significantly decreased odds of receiving a hysterectomy with older age (odds ratio (OR) = 0.98, 95% confidence interval (CI) = 0.97-0.98, p < 0.001), and Black compared to non-Hispanic White race/ethnicity (OR = 0.40, 95% CI = 0.33-0.47, p < 0.001). Median county income of <$45,000 (OR = 0.68, 95% CI = 0.55-0.84, p < 0.001), $45,000-59,999 (OR = 0.83, 95% CI = 0.72-0.96, p = 0.010), and $60,000-74,999 (OR = 0.87, 95% CI = 0.77-0.99, p = 0.030) compared to ≥$75,000 was also significantly associated with decreased odds of receiving a hysterectomy. The odds of receiving a hysterectomy were also significantly lower in those with stage II (OR = 0.12, 95% CI = 0.10-0.15, p < 0.001), III (OR = 0.15, 95% CI = 0.13-0.17, p < 0.001), and IV (OR = 0.05, 95% CI = 0.04-0.07, p < 0.001) compared to stage I cervical cancer.

On adjusted regression analyses, Black patients (OR = 0.46, 95% CI = 0.37-0.56, p < 0.001) were significantly less likely to receive a hysterectomy. Patients with stage II-IV cancer (II: OR = 0.12, 95% CI = 0.10-0.15, p < 0.001; III: OR = 0.15, 95% CI = 0.13-0.17, p < 0.001; IV: OR = 0.05, 95% CI = 0.04-0.07, p < 0.001) were also less likely to undergo the procedure (Table 5).

**Table 5:** Unadjusted and adjusted odds of receiving a hysterectomy. *: The adjusted regression was adjusted for all variables listed in the table.

	Unadjusted		Adjusted*	
	OR (95% CI)	P-value	OR (95% CI)	P-value
Residence				
Urban	(reference)			
Rural	1.08 (0.92-1.28)	0.337	1.07 (0.86-1.33)	0.530
Age	0.98 (0.97-0.98)	<0.001	0.99 (0.99-0.99)	0.044
County median household income				
$75,000	(reference)			
$60,000–74,999	0.87 (0.77-0.99)	0.030	0.93 (0.80-1.08)	0.348
$45,000–59,999	0.83 (0.72-0.96)	0.010	0.97 (0.81-1.17)	0.781
< $45,000	0.68 (0.55-0.84)	<0.001	0.88 (0.68-1.14)	0.335
Race/Ethnicity				
Non-Hispanic White	(reference)			
Hispanic White	0.91 (0.78-1.06)	0.223	0.85 (0.71-1.02)	0.074
Black	0.40 (0.33-0.47)	<0.001	0.46 (0.37-0.56)	<0.001
Other	0.92 (0.78-1.07)	0.269	0.99 (0.82-1.20)	0.936
Stage				
I	(reference)			
II	0.12 (0.10-0.14)	<0.001	0.12 (0.10-0.15)	<0.001
III	0.14 (0.12-0.17)	<0.001	0.15 (0.13-0.17)	<0.001
IV	0.05 (0.04-0.06)	<0.001	0.05 (0.04-0.07)	<0.001

Disparities in overall survival

**Figure 2:**
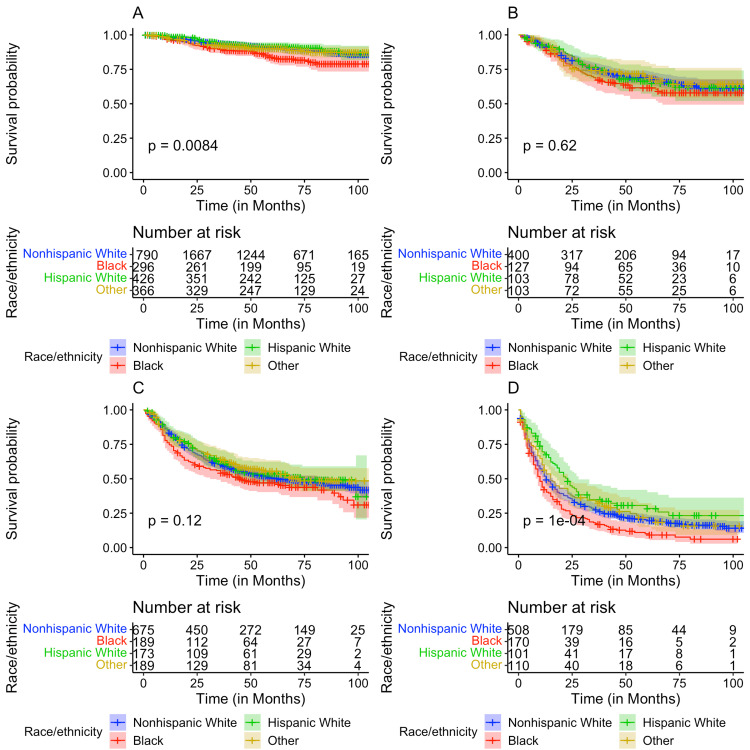
Kaplan-Meier plots comparing overall survival for patients by race/ethnicity with (A) Stage I, (B) Stage II, (C) Stage III, and (D) Stage IV cancer.

**Figure 3:**
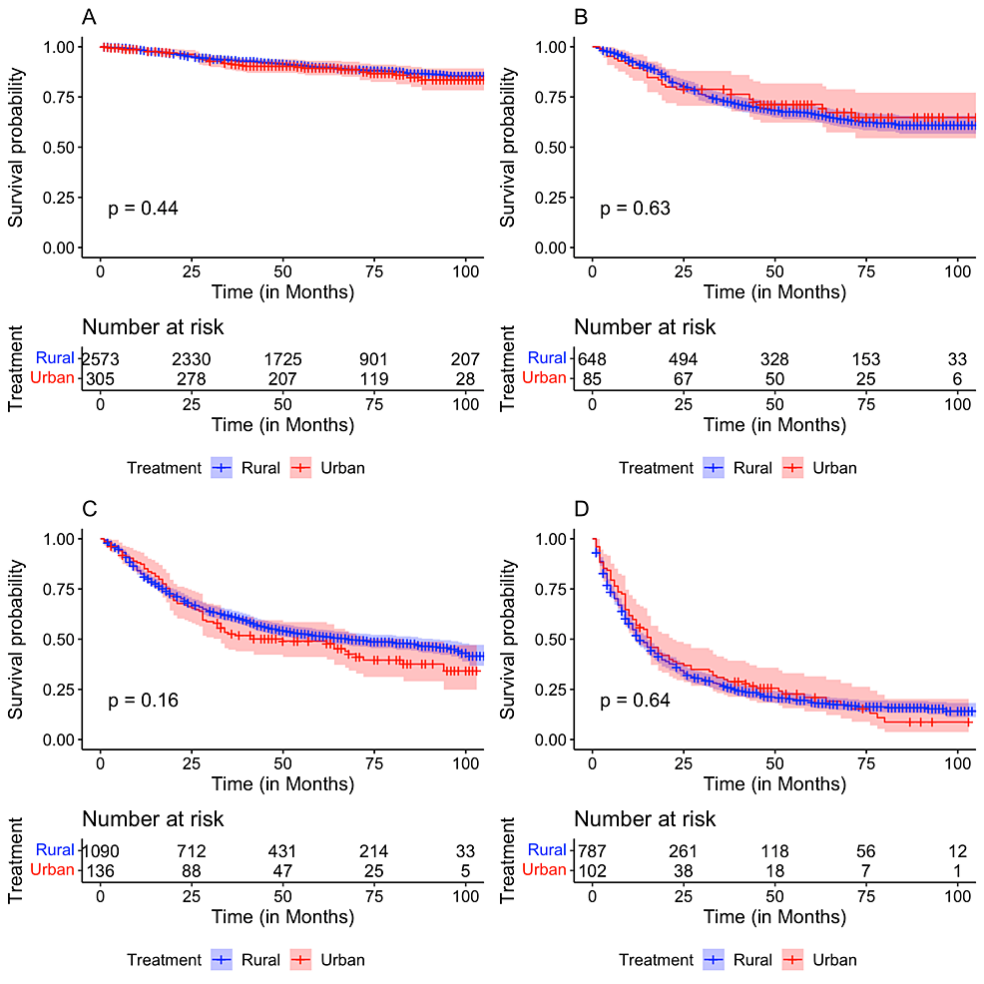
Kaplan-Meier plots comparing overall survival for patients residing in rural versus urban counties with (A) Stage I, (B) Stage II, (C) Stage III, and (D) Stage IV cancer.

**Figure 4:**
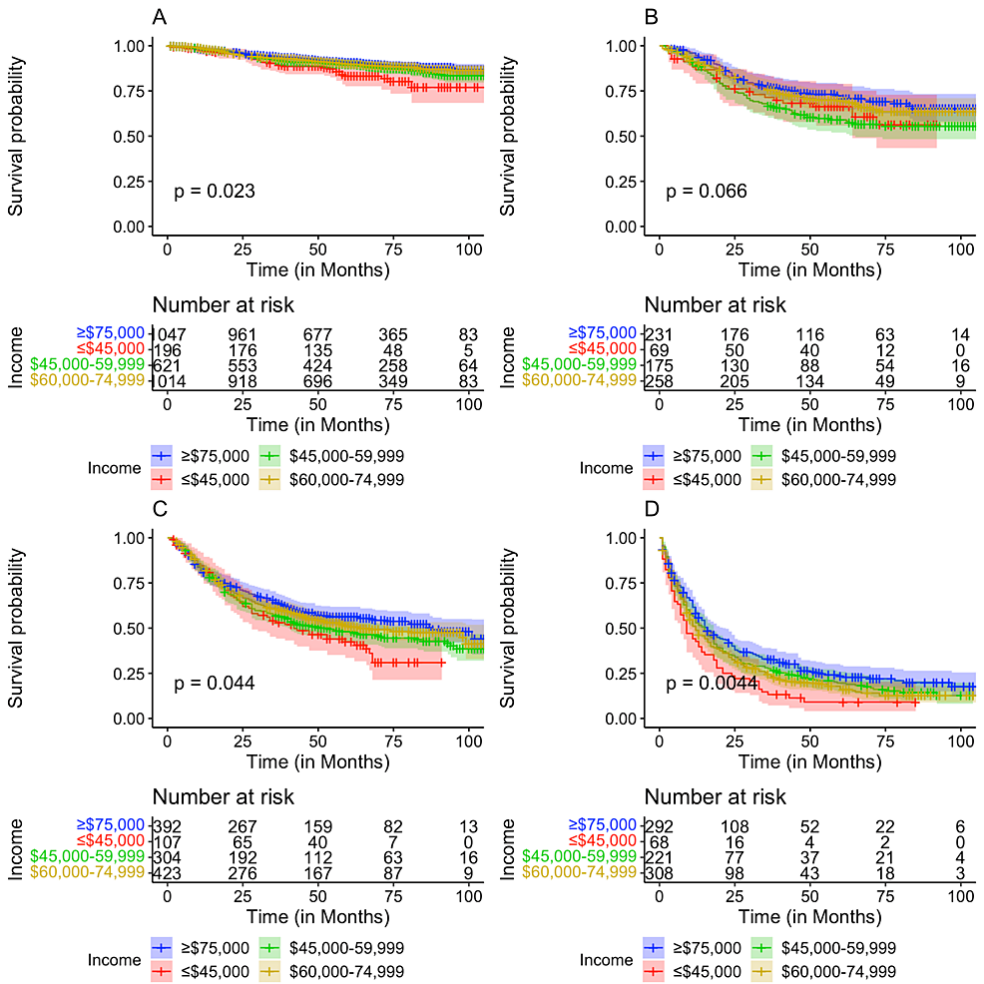
Kaplan-Meier plots comparing overall survival by patient median county income with (A) Stage I, (B) Stage II, (C) Stage III, and (D) Stage IV cancer.

After stratifying by cancer stage at diagnosis, Kaplan-Meier analysis for OS was performed. A significant difference in OS between the race/ethnicities at Stage I (p = 0.0084) and Stage IV (P = 1e-04) cervical cancer was found (Figure 2). A significant difference in OS based on patient median county income at Stage I (p = 0.023), III (p = 0.044), and IV (p = 0.0044) was identified, while the comparison at Stage II was trending toward significance (p = 0.066) (Figure 3). In contrast, no significant difference in OS among patients residing in rural versus urban counties was identified (all p > 0.05) (Figure 4).

Additionally, proportional hazards analyses were performed to quantify the association between the sociodemographics and OS after stratifying by cancer stage and diagnosis. On unadjusted analyses, Stage I, II, III, and IV showed significant differences in OS by different county median household incomes. At Stage I, those who resided in counties with a median household income <$45,000 were found to have significantly poorer OS compared to those with an income ≥$75,000 (HR = 1.81, 95% CI = 1.21-2.72, p = 0.004). An income of $45,000-59,999 was associated with significantly poorer OS compared to ≥$75,000 at stage II (HR = 1.52, 95% CI = 1.09-2.13, p = 0.014) and stage III (HR = 1.46, 95% CI = 1.09-1.95, p = 0.012). At Stage IV, incomes of $60,000-74,999 (HR = 1.25, 95% CI = 1.04-1.50, p = 0.012) and <$45,000 (HR = 1.63, 95% CI = 1.22-2.17, p < 0.001) were found to be associated with significantly poorer OS. Additionally, at Stage I, Black patients were found to have significantly poorer OS compared to non-Hispanic White patients at Stage I (HR = 1.67, 95% CI = 1.21-2.29, p = 0.002) and Stage IV (HR = 1.35, 95% CI = 1.12-1.63, p = 0.001) while Hispanic White patients were also found to have significantly poorer survival compared to non-Hispanic White patients at stage IV (HR = 0.73, 95% CI = 0.56-0.94, p = 0.015) (Table 6).

**Table 6:** Unadjusted (univariate) proportional hazards models for overall survival stratified by cancer stage at diagnosis.

	Stage I		Stage II		Stage III		Stage IV	
	HR (95% CI)	P-value	HR (95% CI)	P-value	HR (95% CI)	P-value	HR (95% CI)	P-value
Age	1.06 (1.05-1.07)	<0.001	1.03 (1.02-1.04)	<0.001	1.02 (1.02-1.03)	<0.001	1.02 (1.01-1.03)	<0.001
Residence								
Urban	(reference)		(reference)		(reference)		(reference)	
Rural	1.14 (0.81-1.61)	0.442	0.91 (0.61-1.35)	0.630	1.19 (0.93-1.51)	0.163	0.95 (0.75-1.19)	0.626
County median household income								
$75,000	(reference)		(reference)		(reference)		(reference)	
$60,000–74,999	1.08 (0.82-1.42)	0.605	1.12 (0.81-1.56)	0.503	1.10 (0.90-1.34)	0.372	1.25 (1.04-1.50)	0.012
$45,000–59,999	1.25 (0.93-1.70)	0.142	1.52 (1.09-2.13)	0.014	1.24 (1.00-1.53)	0.054	1.17 (0.96-1.42)	0.124
< $45,000	1.81 (1.21-2.72)	0.004	1.38 (0.87-2.19)	0.175	1.46 (1.09-1.95)	0.012	1.63 (1.22-2.17)	<0.001
Race/Ethnicity								
Non-Hispanic White	(reference)		(reference)		(reference)		(reference)	
Hispanic White	0.89 (0.62-1.29)	0.540	1.01 (0.69-1.49)	0.957	0.94 (0.73-1.21)	0.691	0.73 (0.56-0.94)	0.015
Black	1.67 (1.21-2.29)	0.002	1.23 (0.89-1.71)	0.214	1.24 (1.00-1.55)	0.051	1.35 (1.12-1.63)	0.001
Other	1.04 (0.73-1.47)	0.820	0.97 (0.66-1.44)	0.891	0.91 (0.72-1.16)	0.461	0.91 (0.72-1.15)	0.424
Hysterectomy								
No	(reference)		(reference)		(reference)		(reference)	
Yes	0.32 (0.25-0.40)	<0.001	0.63 (0.45-0.89)	0.010	0.42 (0.34-0.52)	<0.001	0.40 (0.31-0.53)	<0.001

Following univariate analyses, multivariable proportional hazards analyses were performed after stratifying by cancer stage at diagnosis. Significant differences in survival by different county median household incomes were identified at Stages I, II, and IV. At Stage I, residing in a county with an average household income <$45,000 compared to ≥$75,000 was associated with significantly poorer OS (HR = 1.53, 95% CI = 1.00-2.33, p = 0.048). At Stage II, income between $45,000-59,999 compared to ≥75,000 was also associated with a significantly poorer OS (HR = 1.67, 95% CI = 1.19-2.35, p = 0.003). At Stage IV, income <$45,000 (HR = 1.64, 95% CI = 1.22-2.19, p < 0.001) and income between $60,000-74,999 (HR = 1.23, 95% CI = 1.01-1.48, p = 0.035) were significantly associated with poorer OS compared to ≥$75,000. In addition, at Stage IV, Black compared to non-Hispanic white race/ethnicity was significantly associated with poorer OS (HR = 1.29, 95% CI = 1.06-1.56, p = 0.009) (Table 7).

**Table 7:** Adjusted (multivariable) proportional hazards models for overall survival stratified by cancer stage at diagnosis.

	Stage I		Stage II		Stage III		Stage IV	
	HR (95% CI)	P-value	HR (95% CI)	P-value	HR (95% CI)	P-value	HR (95% CI)	P-value
Age	1.06 (1.05-1.06)	<0.001	1.03 (1.02-1.04)	<0.001	1.02 (1.01-1.03)	<0.001	1.02 (1.01-1.02)	<0.001
County median household income								
$75,000	(reference)		(reference)		(reference)		(reference)	
$60,000–74,999	1.25 (0.93-1.66)	0.136	1.17 (0.84-1.63)	0.349	1.04 (0.85-1.29)	0.689	1.23 (1.01-1.48)	0.035
$45,000–59,999	1.30 (0.95-1.78)	0.104	1.67 (1.19-2.35)	0.003	1.22 (0.98-1.53)	0.075	1.15 (0.94-1.41)	0.174
< $45,000	1.53 (1.00-2.33)	0.048	1.50 (0.94-2.38)	0.087	1.35 (1.00-1.83)	0.051	1.64 (1.22-2.19)	<0.001
Race/Ethnicity								
Non-Hispanic White	(reference)		-	-	(reference)		(reference)	
Hispanic White	1.09 (0.75-1.58)	0.657	-	-	0.98 (0.76-1.26)	0.846	0.82 (0.63-1.07)	0.146
Black	1.08 (0.78-1.51)	0.646	-	-	1.15 (0.91-1.43)	0.236	1.29 (1.06-1.56)	0.009
Other	1.20 (0.84-1.73)	0.320	-	-	0.94 (0.73-1.20)	0.610	0.94 (0.74-1.20)	0.607
Hysterectomy								
No	(reference)		(reference)		(reference)		(reference)	
Yes	0.33 (0.26-0.42)	<0.001	0.68 (0.48-0.95)	<0.001	0.46 (0.37-0.57)	<0.001	0.43 (0.32-0.56)	<0.001

## Discussion

The present analysis of a national, population-based oncology registry revealed significant sociodemographic disparities in cervical cancer patient diagnosis, treatment, and prognosis. Black patients and patients residing in counties with lower median household incomes were more frequently diagnosed with higher cancer stages. On adjusted analysis, Black patients were also found to have significantly decreased odds of receiving a hysterectomy as well as significantly poorer OS for those diagnosed with stage IV cervical cancer. Furthermore, patients residing in counties with lower median household incomes were found to have significantly poorer OS when diagnosed at Stages I, II, and IV.

In our study, Black and Hispanic White women were less frequently diagnosed with Stage I cervical cancer compared to non-Hispanic White women. Several studies support these findings and elaborate on the disparity of cancer staging at diagnosis for racial and ethnic minorities, citing disparities in access to healthcare and lack of screening as potential causes [17,18]. Overall survival was also decreased for these patients, which aligns with the fact that earlier diagnosis in non-Hispanic White women allows for improved outcomes. It is also worth noting that hysterectomy is typically recommended for early-stage cancer but is not necessarily indicated in late-stage cancers, which might explain lower rates of hysterectomy in African American and Hispanic women in light of our results on staging at the time of diagnosis [19].

The differences in disease staging at diagnosis are likely multifactorial but are potentially heavily influenced by patient sociodemographics. In the current literature, there is a known negative correlation between socioeconomic status and risk of malignancy. Previous studies have demonstrated that cervical cancer incidence and mortality can vary by as much as 10-20× between countries, possibly attributed to social inequities [20]. Factors such as low levels of education can lead to decreased participation in routine cancer screenings, resulting in later diagnoses for these patients. Similarly, a lack of resources (lack of sick leave, transportation, etc.) can also compromise individuals’ ability to seek preventative care [20]. Singh et al. emphasized that lack of education and limited access to resources are two substantial barriers responsible for the underutilization of cervical cancer screenings and overall poorer prognoses [20].

Our analysis also revealed a lower likelihood of receiving a hysterectomy for older patients and Black patients. Past research has shown that hysterectomy incidence increases with age, as patients over 60 years had a higher incidence than those under 60 years [21]. Beavis et al. supported this, reporting that the peak age for hysterectomy was 65-69 years [13]. It is possible that treatment protocols vary across healthcare systems and geographic locations, or that our patients’ beliefs and health practices differed from those in other studies. Older patients also tend to be poorer candidates for surgery. Matz et al. described that cervical cancer screening is not always recommended for patients older than 65 years if previous examinations were negative, but cancer incidence does increase until 85 years [17]. This gap accounts for the fact that older women who are not screened and diagnosed with cervical cancer are unlikely to receive treatment, which may explain the negative association between receipt of a hysterectomy and increasing age. Furthermore, Del Carmen et al. described in their study that white women received hysterectomies 76% of the time compared to 9% for African American women [22]. These findings are attributed to an earlier diagnosis of cervical cancer, averaging at Stage IA, while the average African American woman was diagnosed at Stage II. This may account for part of the racial disparity in treatment and prognosis, as Black women more often received radiation therapy, while White women more often received hysterectomies [22].

The poorer OS among Black women compared to non-Hispanic White women with cervical cancer that was identified in our analysis is widely reported in the current literature. Beavis et al. reported that the mortality rate for Black women with cervical cancer is 10.1 per 100,000, compared to 4.7 per 100,000 for White women [13]. They attributed this disparity to several factors including inadequate screening for African Americans due to lower access to healthcare and improper treatment of the cancer [13]. Other factors that negatively impact the cervical cancer survival rate for African American women include unconscious bias, delay in treatment, and miscommunication between the patient and healthcare provider [23]. Each of these factors, in combination with decreased rates of hysterectomy in African Americans, contributes to the broad disparity in mortality and, ultimately, overall cervical cancer outcomes between African American and White women.

Our study is not without limitations. Patients with incomplete data for any variables of interest were omitted from the study sample, which may subject the cohort to selection bias. Patients who were lost to follow-up for OS analyses may also contribute to selection bias, as access to care, lack of transportation, and positive response to treatment may discourage patients from reconvening with their physicians [24,25]. Given the use of SEER, variables that could be included in the analysis were limited to those available in the database. Sociodemographic variables such as patient insurance status and educational level could not be assessed. Administration of treatments such as chemotherapy and outcomes including disease recurrence were similarly unable to be included in our analyses. The lack of data on patients’ adherence to routine screening and preventative measures, such as Pap smears or HPV vaccination status, also limits our interpretation of the results. Additionally, the potential for miscoding of ICD codes in the database should be acknowledged.

## Conclusions

The current study from a national, population-based database demonstrated notable inequities in diagnosis and prognosis for women with cervical cancer based on sociodemographic groups. Our analysis indicated that Black patients were more often diagnosed with greater disease progression, were less likely to receive a hysterectomy, and had poorer OS compared to other race/ethnicity groups. Additionally, patients residing in a county with a lower median household income were similarly more often diagnosed with greater disease progression and were found to have poorer OS compared to more affluent counties. These findings may help guide policymakers in developing strategies to improve access to preventative medicine and reduce inequities in care for these marginalized groups.
